# Supplementary material for: Single Laboratory Evaluation of the Q20+ Nanopore Sequencing Kit for Bacterial Outbreak Investigations
Source: Int J Mol Sci. 2024 Nov 5;25(22):11877. doi: 10.3390/ijms252211877 (PMC11594029; doi:10.3390/ijms252211877)
Supplement: Supplementary file 1 [file ijms-25-11877-s001.zip › ijms-3257365-supplementary.pdf]

Supplementary Materials

Supplementary Figures

Supplementary Figure S1. Schematic flow chart for the proposed comparison in this study of the two DNA extraction methods (sheared and intact DNA) and the two DNA library preparations (Ligation and rapid kit)

Supplementary Figure S2. Statistics regarding N50 Length and read numbers per run for the initial testing.

Supplementary Figure S3. Complete NJ tree of the wgMLST analyses for CFSAN000189**.**

Supplementary Figure S4. Complete NJ tree of the wgMLST analyses for CFSAN123154**.**

Supplementary Figure S5. Complete NJ tree of the wgMLST analyses for CFSAN030807**.**

Supplementary Figure S6. Complete NJ tree of the wgMLST analyses for CFSAN086181**.**

Supplementary Figure S1. Schematic flow chart for the proposed comparison in this study of the two DNA extraction methods (sheared and intact DNA) and the two DNA library preparations (Ligation and rapid kit).

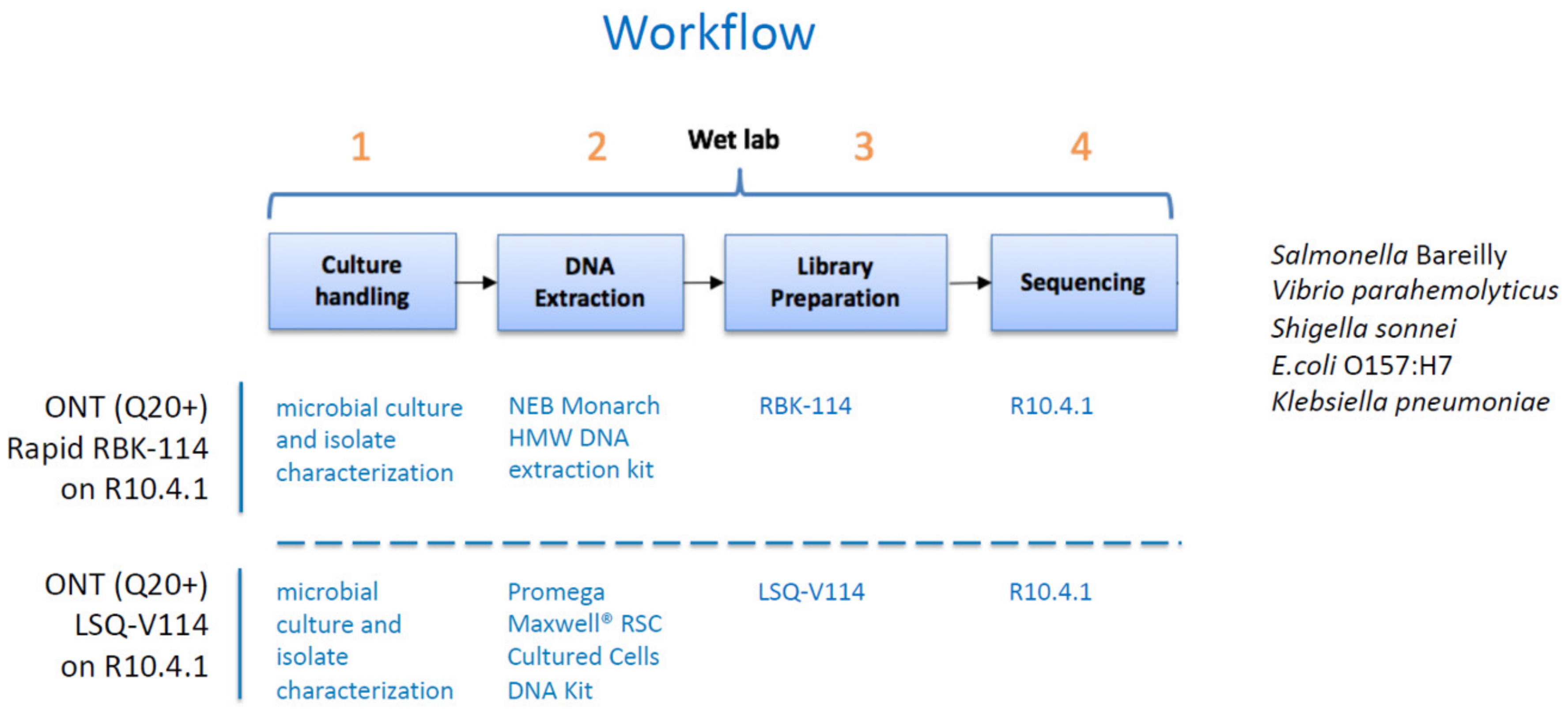

Supplementary Figure S2. Statistics regarding N50 Length and read numbers per run for the initial testing.

A) B)

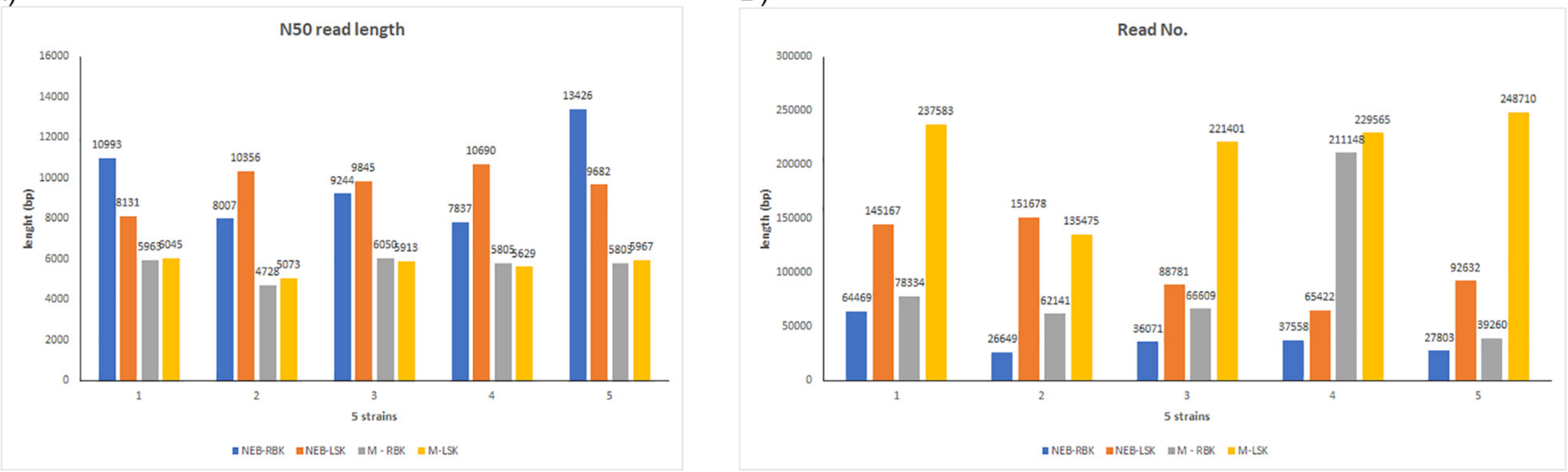

Supplementary Figure S3. Complete NJ tree of the wgMLST analyses for CFSAN000189**.**

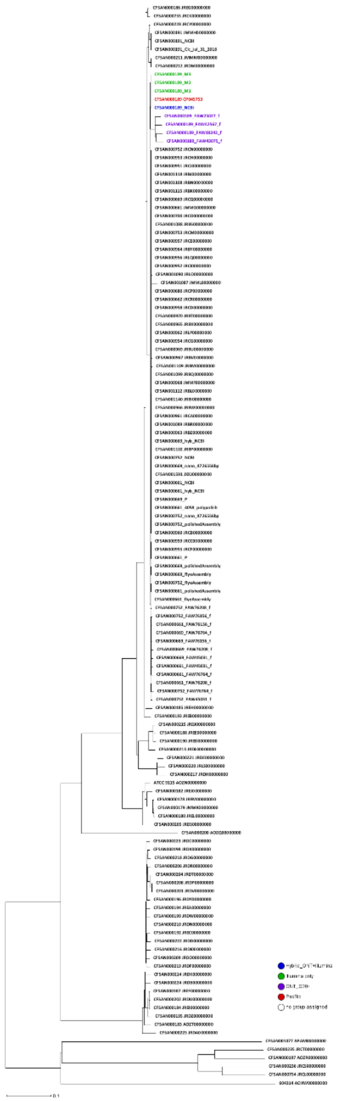

Supplementary Figure S4. Complete NJ tree of the wgMLST analyses for CFSAN123154**.**

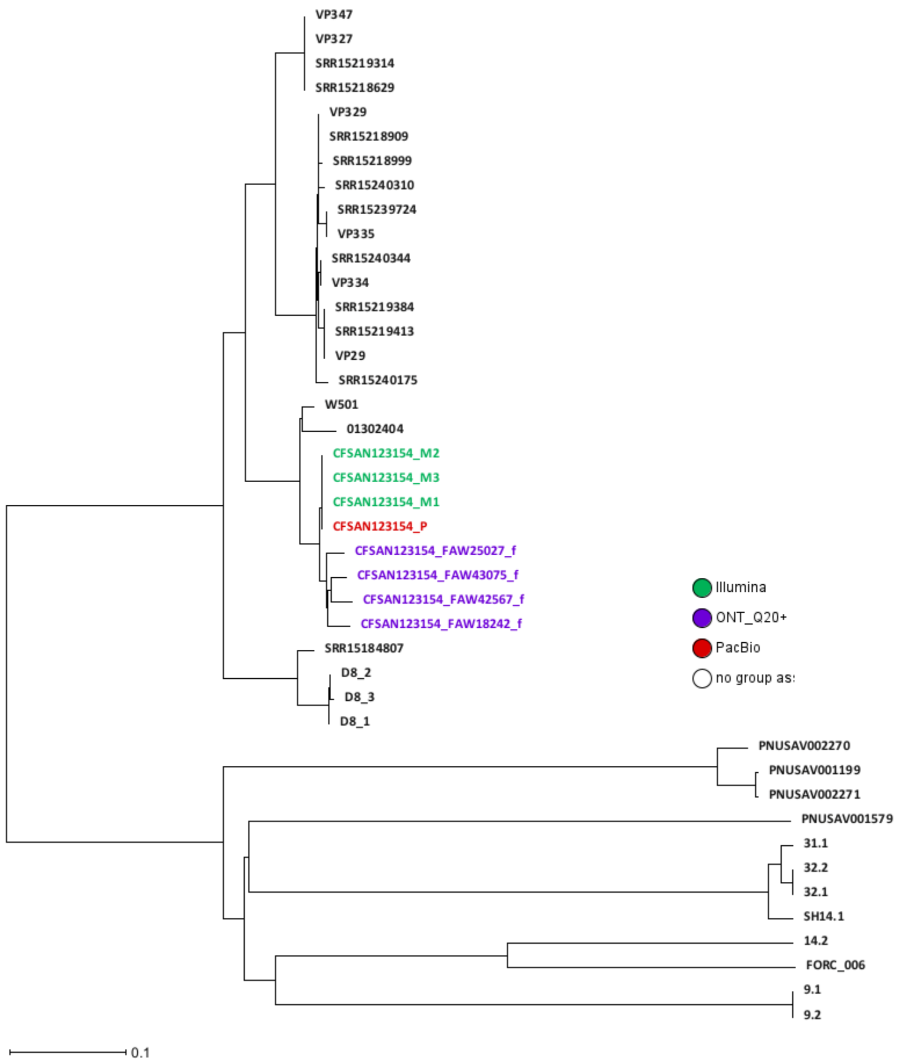

Supplementary Figure S5. Complete NJ tree of the wgMLST analyses for CFSAN030807**.**

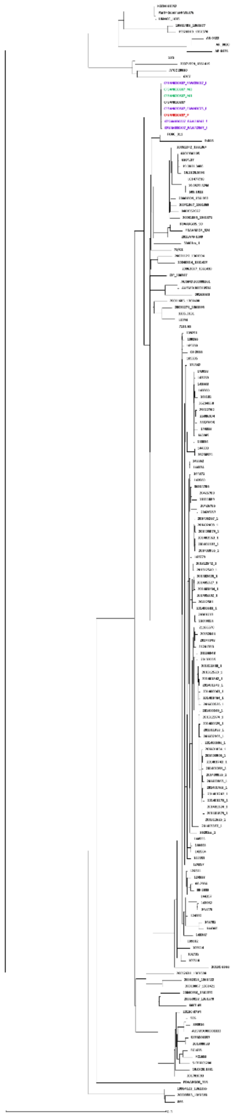

Supplementary Figure S6. Complete NJ tree of the wgMLST analyses for CFSAN086181**.**

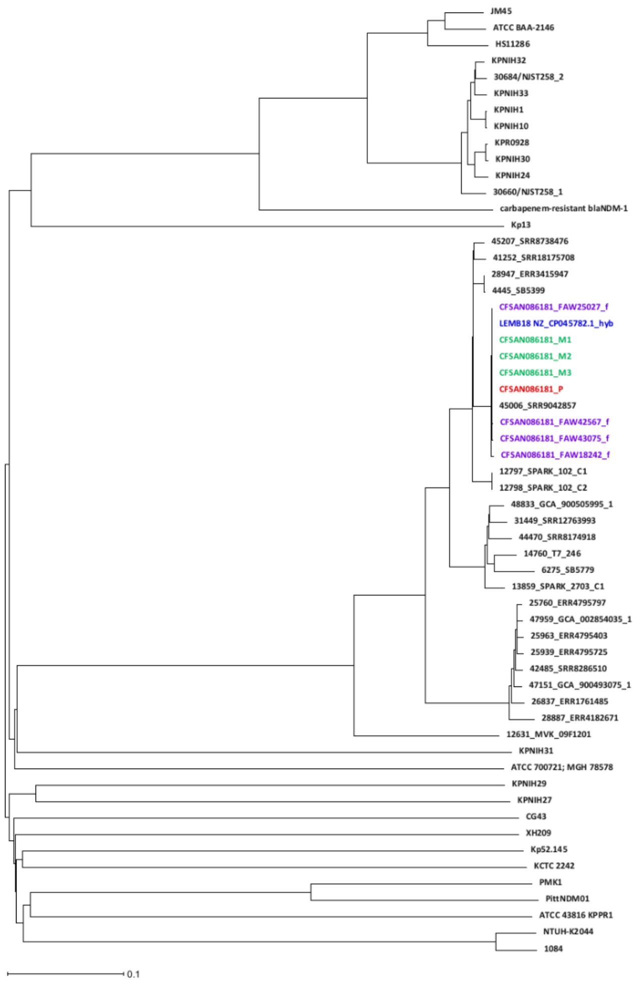

Supplementary Table S1. SRA data deposited at the Genbank for each sample and experiment (DNA extraction and library combination).

CFSAN No.	Strain	DNA extraction method	DNA library preparation method	SRA accession No.	Flow cell No.
CFSAN000189	*Salmonella enterica* subsp. *enterica* serotype Bareilly	Maxwell	RBK114	SRR29782658	FAW25027
CFSAN123154	*Vibrio parahemolyticus*	Maxwell	RBK114	SRR29782657	FAW25027
CFSAN030807	*Shigella sonnei*	Maxwell	RBK114	SRR29782656	FAW25027
CFSAN076620	*Escherichia coli*	Maxwell	RBK114	SRR29782655	FAW25027
CFSAN086181	*Klebsiella pneumoniae*	Maxwell	RBK114	SRR29782654	FAW25027
CFSAN000189	*Salmonella enterica* subsp. *enterica* serotype Bareilly	Maxwell	LSK-114	SRR29782678	FAW43075
CFSAN123154	*Vibrio parahemolyticus*	Maxwell	LSK-114	SRR29782677	FAW43075
CFSAN030807	*Shigella sonnei*	Maxwell	LSK-114	SRR29782666	FAW43075
CFSAN076620	*Escherichia coli*	Maxwell	LSK-114	SRR29782660	FAW43075
CFSAN086181	*Klebsiella pneumoniae*	Maxwell	LSK-114	SRR29782659	FAW43075
CFSAN000189	*Salmonella enterica* subsp. *enterica* serotype Bareilly	NEB	RBK114	SRR29782671	FAW18242
CFSAN123154	*Vibrio parahemolyticus*	NEB	RBK114	SRR29782670	FAW18242
CFSAN030807	*Shigella sonnei*	NEB	RBK114	SRR29782669	FAW18242
CFSAN076620	*Escherichia coli*	NEB	RBK114	SRR29782668	FAW18242
CFSAN086181	*Klebsiella pneumoniae*	NEB	RBK114	SRR29782667	FAW18242
CFSAN000189	*Salmonella enterica* subsp. *enterica* serotype Bareilly	NEB	LSK-114	SRR29782676	FAW42567
CFSAN123154	*Vibrio parahemolyticus*	NEB	LSK-114	SRR29782675	FAW42567
CFSAN030807	*Shigella sonnei*	NEB	LSK-114	SRR29782674	FAW42567
CFSAN076620	*Escherichia coli*	NEB	LSK-114	SRR29782673	FAW42567
CFSAN086181	*Klebsiella pneumoniae*	NEB	LSK-114	SRR29782672	FAW42567
CFSAN000189	*Salmonella enterica* subsp. *enterica* serotype Bareilly	Maxwell	RBK114	SRR29782665	FAW81132

CFSAN123154	*Vibrio parahemolyticus*	Maxwell	RBK114	SRR29782664	FAW81132
CFSAN030807	*Shigella sonnei*	Maxwell	RBK114	SRR29782663	FAW81132
CFSAN076620	*Escherichia coli*	Maxwell	RBK114	SRR29782662	FAW81132
CFSAN086181	*Klebsiella pneumoniae*	Maxwell	RBK114	SRR29782661	FAW81132

Maxwell- Maxwell RSC Cultured Cell DNA kit. RBK114 - SQK-RBK114.24 kit, NEB- Monarch high molecular weight (HMW). LSK114 - SQK-LSK114 kit.
